# An *in-vitro* assay using human spermatozoa to detect toxicity of biologically active substances

**DOI:** 10.1038/s41598-019-50929-z

**Published:** 2019-10-10

**Authors:** Tino Vollmer, Börje Ljungberg, Vera Jankowski, Joachim Jankowski, Griet Glorieux, Bernd G. Stegmayr

**Affiliations:** 1 Department of Public Health and Clinical Medicine, Umea University, Umea, Sweden; 2 Institute of Medical Immunology, Charité - Universitätsmedizin Berlin, Berlin, Germany; 3 Berlin Institute of Health Center for Regenerative Therapies (BCRT), Berlin-Brandenburg School for Regenerative Therapies (BSRT) & Berlin Center for Advanced Therapies (BeCAT), Charité - Universitätsmedizin Berlin, Berlin, Germany; 4 Department of Surgical and Perioperative Sciences, Urology and Andrology, Umea University, Umea, Sweden; 5 Institute for Cardiovascular Research, University Hospital, RWTH Aachen University, Aachen, Germany; 6 School for Cardiovascular Diseases, University of Maastricht, Maastricht, The Netherlands; 7 Nephrology Section, Department of Internal Medicine and Pediatrics, Ghent University Hospital, Ghent, Belgium

**Keywords:** Biological models, Mechanisms of disease

## Abstract

Identifying the key toxic players within an *in-vivo* toxic syndrome is crucial to develop targeted therapies. Here, we established a novel method that characterizes the effect of single substances by means of an *ex-vivo* incubation set-up. We found that primary human spermatozoa elicit a distinct motile response on a (uremic) toxic milieu. Specifically, this approach describes the influence of a bulk toxic environment (uremia) as well as single substances (uremic toxins) by *real-time* analyzing motile cellular behavior. We established the human spermatozoa-based toxicity testing (HSTT) for detecting single substance-induced toxicity to be used as a screening tool to identify *in-vivo* toxins. Further, we propose an application of the HSTT as a method of clinical use to evaluate toxin-removing interventions (hemodialysis).

## Introduction

An *in-vivo* toxicity situation is observed in end-stage kidney disease (ESKD) which significantly contributes to morbidity and mortality of these patients^1^. During the progressive loss of kidney function, uremic substances, that would normally be excreted by the healthy kidney, accumulate *in-vivo*^2,3^. This leads to a myriad of clinical symptoms coined as the uremic syndrome^4^ and subsequent death, unless hemodialysis (HD) is initiated^5^. A thorough understanding of the biological activity of uremic substances is crucial to unveil mechanisms behind the uremic syndrome.

To investigate the biochemical impact of substances, animal models or cell cultures can be applied^6–9^. Compared to animal models, human *in-vitro* models are more consistent in predicting clinical outcome^10,11^ and more suitable for identifying mechanisms relevant for *in-vivo* toxicity^12^. Recently developed organ-on-a-chip models underline the potential of human-derived system^6,11,13,14^ but a lack of primary cell material and the complexity of recreating a toxic environment in the chip-system limits its application for broad toxicity testing. As an alternative, we established a cellular method derived from an available human cell source in order to mimic a toxic *in-vivo* situation.

Human spermatozoa are a neglected primary cell type of study, however, there is abundant cellular material of human spermatozoa that can be gained from fertile men. Due to the specific function to fertilize an oocyte, spermatozoa have adapted cellular characteristics. E.g. transcriptomic and translational activity are virtually absent in spermatozoa^15,16^ and thus, cell function is mainly regulated on protein level. Hence, due to the inability to produce proteins, spermatozoa rely on the environment for external regulation^17,18^.

By means of mass spectrometry, the proteome of human spermatozoa has been thoroughly characterized^19–21^. Most recent advanced *high-throughput* proteomic approaches resulted into the identification of around 6000 proteins expressed by human spermatozoa^22,23^, wherein redundant pathways of energy metabolism (carbohydrates, lipids, proteins) and apoptosis were significantly represented^16,24^. This makes spermatozoa a functionally defined cell type that strongly resembles the energy apparatus that maintains the cell survival of the human body^25^. These cellular characteristics are ideal for the investigation of substances causing specific effects on the metabolism and the proteomic stability of the cells.

Intriguingly, uremic men have reduced fertility and sperm quality that is assumed to be due to abnormalities of endocrine factors^26^, however toxic effects of *external* uremic toxins cannot be excluded. In line with this, progressive motile function of spermatozoa is impaired in ESKD compared to earlier stages of chronic kidney disease^27^.

Progressive motility defines the capacity of sperm to efficiently move which is required for male fertility^28^. Motile function of human spermatozoa is energetically highly conserved by the ability to switch between mitochondrial oxidative phosphorylation and glycolysis upon the availability of oxygen and substrates^17,29,30^. In contrast to progressive motility, directional motility (chemotaxis) of spermatozoa is induced by cell-specific differentiation stimuli *in-vitro*^31,32^. Thus, progressive motility of spermatozoa can be maintained *in-vitro* that is non-directional and robust against slight environmental alterations but, as we hypothesized, sensitive for *in-vivo* toxins. To investigate *ex-vivo* spermatozoon function, motility can be observed via light microscopy^33^ which facilitates the implementation into an applicable test system.

Uremic toxins reported in the literature have been recently summarized and thereby, the level of evidence has been scored revealing defined organ systems affected by distinct substances^9^. Here, we present an approach that defines toxicity by comparing the effect of many substances within one single cell model. The precise aim of this study was to develop a bio-assay that can be used to analyze toxicity of biological substances in an *in-vitro* setting. Due to the spermatozoon source of the cell model, this may give additional guidance to the identification of toxins that interfere with male fertile function in ESKD.

In a first step, we set up an *ex-vivo* incubation profile of spermatozoa to measure changes in motile function. Second, we screened on the sensitivity of the *in-vitro* model for a bulk uremic milieu. Third, we screened on the sensitivity of the *in-vitro* model for single uremic substances and drugs. Fourth, we applied the newly established human spermatozoa-based toxicity testing (HSTT) to estimate the removal of toxins during an intervention such as hemodialysis in ESKD patients.

This effort represents a joined initiative of the European Uremic Toxin Work Group (EUTox) that is dedicated to the identification of novel toxins in the field of uremic toxicity. Here, we describe the precise establishment of a feasible human-specific *in-vitro* tool to unveil toxins potentially relevant for many fields. Knowledge about identified toxins may be used to improve treatment of a toxic syndrome by a specific toxin-targeting approach.

## Results

### Establishment of an *in-vitro* motility analysis of human spermatozoa

**Figure 1:**
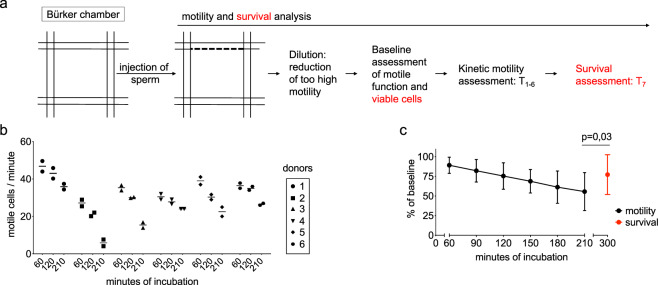
Analysis of motility and survival capacity of spermatozoa *ex-vivo*. **(a)** Assessment of progressively-motile spermatozoa. Dashed, bold line in Bürker chamber indicates the crossing mark for motile cells. **(b)** Defining precision of single-measurement analysis of motile cells. Two dots at each time point show duplicate analysis of one sample. Six donors at three points measured in independent experiments. **(c)** Kinetic assessment of motile function in comparison to survival. Cells analyzed at six time points for motility and thereafter, at one time point for survival. Dots represents mean of six different donors. P-value indicates significance level.

To establish the human spermatozoa-based toxicity testing (HSTT), we set up a method to preserve spermatozoa *ex-vivo*. Standardized clinical methods estimate motile function as an indicator of male fertility^34^. For this, WHO guidelines recommend a snap-shot discrimination into progressively motile, non-progressively motile and immotile cells^28,34^. This allows a clinical analysis of motile function but is, however, not suitable for kinetic assessment of sperm motility over time. To overcome this, a previously used method^35^ was translated into a clinically applicable tool for toxicity testing (Fig. 1a). First, our goal was to set up a precise analysis of human spermatozoa in non-toxic conditions by means of camera-integrated light microscopy (see methods). For this, *ex-vivo* incubation of spermatozoa was performed to investigate motile function. To evaluate the precision of single time-point analyses within the model, duplicate measurements were performed. Here, we found minor changes between two motility measurements of a single sample (difference in motility counts at 60 min 3.6% ± 1.1%, at 120 min 2.5% ± 1.7% and at 210 min 2.6% ± 1.8%) (Fig. 1b). Further, we kinetically analyzed motility function and compared it to the viability of spermatozoa. We could detect linearly decreasing but sustained motile function over time (55.6% ± 24.2% of initial motility after 210 min) (Fig. 1c). In addition, baseline survival was maintained at a significantly higher level over time than baseline motility (77.2% ± 25.4% of initial survival; p = 0.03) (Fig. 1c). In summary, we established a feasible *ex-vivo* method that precisely estimates the decline of motile human function over time. Thereby, viability of spermatozoa can be preserved.

### Detecting toxicity of uremic ultrafiltrate (UUF)

**Figure 2:**
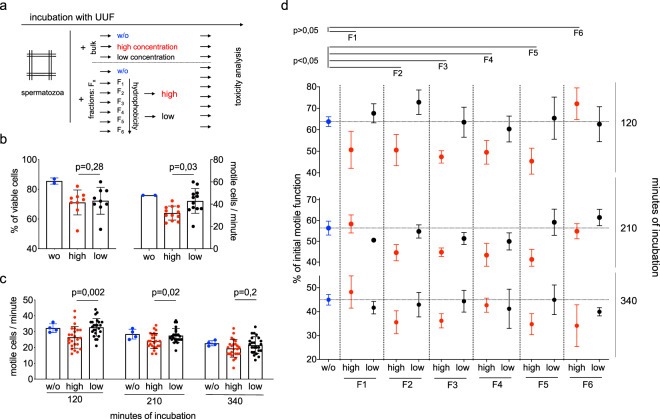
Toxicity detection of uremic ultrafiltrate (UUF). **(a)** Workflow of two experimental approaches. UUF collected from patients during high-flux hemodialysis and prepared by elution into fractions (F). F1 is most hydrophilic and F6 most hydrophobic fraction, respectively. **(b)** High (1:1 dilution of UUF) and low concentration (1:16 dilution of UUF) of bulk UUF were applied in nine independent experiments for viability testing and 12 independent experiments for motility testing. Cells were gained from one donor and incubated for one minute. Two controls (w/o) were applied. **(c)** High and low concentration of UUF tested on motility long-term outcome. 12 independent experiments on cells from two donors, respectively. For each donor, two independent controls were applied (w/o). **(d)** Fractions (F1-F6) tested for toxicity by comparing high (red) vs. low (black) concentration after 120, 210 and 340 minutes of incubation. Mean shows two experimental series on cells from two donors on two conditions (high and low), respectively. Doubled controls were applied for each series (w/o, blue). Tickled line shows medium value of internal control. Bars in **(d)** present SEM. P-value indicates significance level.

Next, the HSTT was applied to detect toxicity of the uremic milieu (Fig. 2a). For this, we accessed uremic ultrafiltrate (UUF) from ESKD patients. UUF has been previously studied as a model of uremic toxicity^36^. Here, we identified a dose-dependent decrease of motility of human spermatozoa when incubated with bulk UUF for one minute (high concentration: 32.0 ± 6.3 vs. low concentration: 42.9 ± 11.1 motile cells/minute, p = 0.03). However, we could not find a significant dose-dependent effect on viability (high concentration: 72.2% ± 9.1% vs. low concentration: 71.1% ± 8.4% of viable cells, p = 0.03) (Fig. 2b). To investigate toxic effects over time, incubation time was extended. Here, we found a complementary dose-dependent decrease of motile function after 120 min (−16.2% ± 30.6%, p = 0,002) and 210 min (−10.5% ± 21.0%, p = 0.02) of incubation, respectively. After 340 min, the cell model could not significantly discriminate between high and low concentrations of UUF (Fig. 2c). This implicates a sensitive time range of the cell model. Further, we tested whether the cells are sensitive for hydrophobic or hydrophilic characteristics of the uremic milieu. For this, elution through a preparative reversed phase column was applied to separate the UUF into six fractions. Within the UUF-fractions, fraction 1 is characterized as the most hydrophilic and contrastingly, fraction 6 as the most hydrophobic part. In a kinetical manner, UUF-fractions were compared for toxicity on motile function. Here, we found lower motility induced by fractions 2–5 compared to the non-toxic control (−21.6% ± 4.1%; p < 0.05). However, we could not detect significant functional alteration induced by the most hydrophilic fraction 1 (−3,8% ± 22.4; p > 0,05) and the most hydrophobic fraction 6 (−3.9% ± 29.0; p > 0.05) (Fig. 2d), respectively. We conclude that the established *in-vitro* model is sensitive for changes of uremic concentration derived from specific fractions within the UUF.

### Detecting toxicity of a uremic toxin mix and drugs

**Figure 3:**
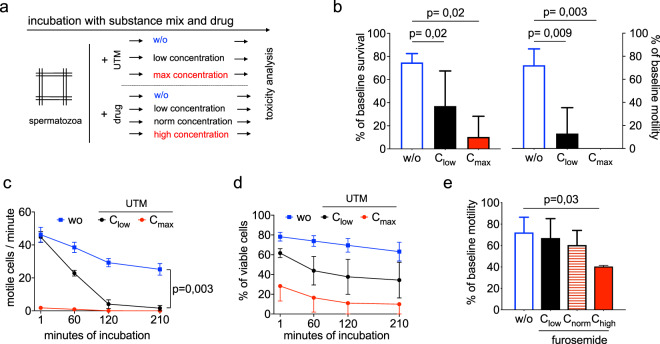
Toxicity detection of substance mix and drug. **(a)** Workflow to measure effects of a substance mix and drugs. Uremic toxin mix was applied in C_max_ (highest concentration measured in uremic patients) and in C_low_ (1:16 dilution of C_max_). Following substances and concentrations were applied within the UTM: Uric acid (C_max_: 147 µg/ml; C_low_: 9.19 µg/ml), xanthine (C_max_: 3.44 µg/ml; C_low_: 0.22 µg/ml), uridine (C_max_: 32.6 µg/ml; C_low_: 2,04 µg/ml) and uracil (C_max_: 0,45 µg/ml; C_low_: 0.03 µg/ml). **(b)** Survival (left) and motility (right) were analyzed after 120 minutes of incubation on a minimum of 3 donors. **(c)** Motility analysis of five experimental rounds. Cells from three different donors. (**d**) Frequency of viable cells over time on cells from three donors. In (**c**) and (**d**) SEM are shown. (**e**) Furosemide was applied to the cell model to test for motility. Incubation of 120 minutes was performed.P-value indicates significance level.

In a next step, the HSTT was used to screen for toxicity of a uremic substance mix (Fig. 3a**)**. For this, we generated a uremic toxin mix (UTM) based on purine derivatives (uric acid, xanthine, uridine, uracil) that were previously shown to co-elute with early fractions of the UUF^36^. Serum concentrations of uremic toxins were applied according to the EUTox database that includes 130 uremic substances^37^. For each toxin, the highest serum concentration reported in uremic patients was installed to the *in-vitro* system and referred to as maximum concentration (C_max_)^3^. Here, we incubated the cells with the UTM to evaluate effects on sperm viability and motility. Strikingly, we found a dose-dependent decrease of motility induced by the UTM. In addition, viability of the cells was diminished by diluted UTM (UTM: 37.0% ± 30.4% vs. w/o: 74.8% ± 7.8%, p = 0.02). When C_max_ of the UTM was applied, motile function was completely abolished, however, a fraction of cells survived (12.2% ± 15.1%) (Fig. 3b). This implicates that motile function is more sensitive for toxicity, whereas viability can be better maintained in the same toxic condition. In a kinetical manner, we could confirm a stringent dose-dependent effect on motility and survival by the UTM (Fig. 3c,d). Further, we found specifically uracil within the UTM to be toxic on the cells (Suppl. Fig. 2). Next, we tested *in-vitro* toxicity of the diuretic drug furosemide that is commonly administered to ESKD patients^38^. For this, furosemide was installed to the cell model and tested for toxic effects. Here, we found a dose-dependent decrease of motility by furosemide (w/o: 72.2% ± 14.2%; C_low_: 66.9% ± 18.2%; C_norm_: 60.5% ± 13.6%; C_high_: 40.3% ± 1.1%; p = 0.03) (Fig. 3e). However, no effect of furosemide on cell survival was observed (*data not shown*). We conclude that the HSTT can be applied to detect toxicity from a mix of uremic substances such as the UTM. In addition, clinically relevant medication can be likewise investigated on potential toxicity.

### Detecting toxicity of single uremic substances

**Figure 4:**
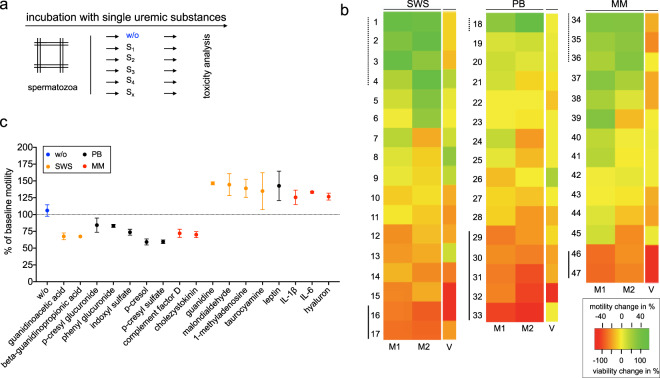
Toxicity detection of denominated uremic toxins (UT). **(a)** Workflow to measure effects of single substances (S_1_-S_x_)**. (b)** 47 UT tested for influence on motility (after 90 minutes of incubation) and survival (after 300 minutes of incubation). UT are assigned to the three bio-chemical classes (small water-soluble compounds (SWS), protein bound (PB), middle molecules (MM)). Data are shown as relative change to non-toxic control. M1 and M2 represent two separate experimental motility rounds, V represents mean viability of six experimental rounds. Bold lines mark UT with reduced motility <25%, dashed lines mark UT with increased motility >25%. Marked UT are displayed in **(c)**. Left: all UT with toxic effect, right: all UT with non-toxic effect. At least two separate experimental rounds are shown. Incubation was performed for 90 minutes.

In a next step, we applied the HSTT to previously denominated uremic toxins (UT). The UT can be separated into three classes along their physico-chemical characteristics: small water-soluble compounds (SWS), protein-bound substances (PB) and middle molecules (MM)^39^. To our knowledge, there is no study comparing multiple substances from all classes within one cell model. To establish this concept, we analyzed 47 UT (Table 1) derived from all three classes via the motility and survival assays of the HSTT (Fig. 4a). Here, we found most profound toxic effects from substances of the protein-bound class (change in function; PB: −10.0% ± 21.1%, SWS: 1.8% ± 26.3%; MM: 5.0% ± 19.0%) (Fig. 4b). In line with this, toxic effects by protein-bound substances have been described^40^. Specifically, we identified toxic effects by the substance group of cresoles and indoles (# **28–33**); e.g. p-cresyl sulfate (#**33**) and indoxyl sulfate (#**32**) (Fig. 4c). Toxicity by these substance groups is confirmed by a recent meta-analysis on UT^9^. Further, we could discriminate toxic (e.g. p-cresyl sulfate, #**33**) from non-toxic substances (e.g. leptin, #**18**) in every substance class, respectively (number of toxic substances/total substances in class; PB: 12/16, SWS: 11/17; MM: 5/14). In addition, we found that motility and viability were likewise abolished by toxic substances (#**15–17**, **31–32**, **46–47**). By this approach, we confirm the toxicity of known UT and further, we can discriminate toxic from non-toxic uremic substances. Serial application of the HSTT may enable the identification of novel candidates that contribute to the uremic syndrome.

**Table 1:** List of uremic toxins tested. C_max_ indicates applied concentration. NaCl or H_2_O were used as solvents. Solvent fraction defines remaining volume (V) of solvent *in-vitro* (by the ratio: V_solvent_/V_total_).

substance	unit	C_max_	substance class	solvent fraction	# in Fig. 4	UT class
guanidine	µg/l	800	guanidines	0.00067797	1	SWS
malondialdehyde	µg/l	769.6	aldehydes	0.00541972	2	SWS
1-methyladenosine	µg/l	216.4	ribonucleosides	0.00006418	3	SWS
taurocyamine	µg/l	121.8	guanidines	0.05000000	4	SWS
methylguanidine	µg/l	1820	guanidines	0.00207763	5	SWS
cytidine	µg/l	1263.6	purines	0.00027000	6	SWS
urea	g/L	4.6	other	0.25000000	7	SWS
orotidine	mg/l	47.2	pyrimidines	0.16666667	8	SWS
N-acetylarginine	µg/l	4580	guanidines	0.01060185	9	SWS
uric acid	mg/l	146.7	purines	0.21830357	10	SWS
SDMA	µg/l	1232.2	guanidines	0.00305000	11	SWS
1-methylguanosine	µg/l	89.2	ribonucleosides	0.00002503	12	SWS
oxalate	mg/l	7.6	other	0.16666667	13	SWS
uracil	µg/l	448	purines	0.00033333	14	SWS
hypoxanthine	mg/l	5.3	purines	0.01948529	15	SWS
guanidinoacetic acid	µg/l	693.8	guanidines	0.00296496	16	SWS
beta-guanidinopropionic acid	µg/l	65.4	guanidines	0.00024962	17	SWS
leptin	µg/l	490	peptides	0.16666625	18	PB
kynurenine	µg/l	952.6	indoles	0.00228990	19	PB
kynurenic acid	mg/l	9.5	indoles	0.02513228	20	PB
methylglyoxal	µg/l	146	AGE	0.16666663	21	PB
phenyl sulfate	mg/l	1.6	phenols	0.01666667	22	PB
2-methoxyresorcinol	µg/l	322	phenols	0.16666625	23	PB
3-deoxyglucosone	mg/l	3.5	AGE	0.16666625	24	PB
homocysteine	mg/l	26.4	amino acid	0.25000000	25	PB
p-OH-hippuric acid	mg/l	31.5	Hippurates	0.08076923	26	PB
putrescine	µg/l	132	Polyamines	0.00007500	27	PB
indoxyl glucuronide	mg/l	3.87	indoles	0.01666667	28	PB
p-cresyl glucuronide	mg/l	5.1	cresoles	0*	29	PB
phenyl glucuronide	mg/l	1.6	phenols	0.00166667	30	PB
indoxyl sulfate	mg/l	236	indoles	0.01666667	31	PB
p-cresol	mg/l	40.7	cresoles	0.18750000	32	PB
p-cresyl sulfate	mg/l	5.1	phenols	0.01666667	33	PB
IL−6	ng/l	328.1	cytokines	0.16666667	34	MM
hyaluron	µg/l	1843	peptides	0.00003686	35	MM
IL-1ß	ng/l	1700	cytokines	0.16666663	36	MM
ß-endorphin	ng/l	492	peptides	0.00000001	37	MM
neuropeptide Y	ng/l	115.9	peptides	0.00000001	38	MM
K-Ig light chain	mg/l	287	peptides	0.25000000	39	MM
retinol binding protein	mg/l	369.2	peptides	0.00000001	40	MM
adrenomedullin	ng/l	81.2	peptides	0.00000007	41	MM
TNF-α	ng/l	408	cytokines	0.00000001	42	MM
endothelin	ng/l	129.4	peptides	0.00000002	43	MM
atrial natriuretic peptide	ng/l	436.6	peptides	0.00000071	44	MM
L-Ig light chain	mg/l	328	peptides	0.00656000	45	MM
complement factor D	mg/l	26	peptides	0.00054737	46	MM
cholecystokinin	ng/l	131.5	peptides	0.16625000	47	MM

PBS was used to dilute. UT = uremic toxins. SWS = small water-soluble compounds, PB = protein bound, MM = middle molecules. AGE = advanced glycation end products. *Directly solved by PBS.

### Estimating toxin-removal by hemodialysis

**Figure 5:**
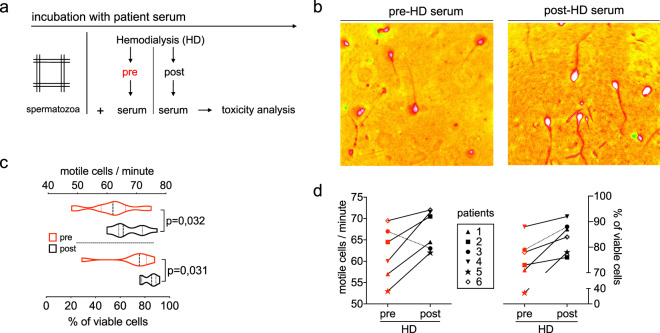
Detection of toxin-removal by hemodialysis. **(a)** Sera from six hemodialysis (HD) patients were taken pre and post HD. Incubation procedure was performed with human spermatozoa. **(b)** Staining of one representative experiment. Eosin^+^ dead cells stained red. White cytoplasm indicates viability. **(c)** Motility counts per minute and viability of cells after 120 minutes of incubation with pre- and post-HD serum, respectively. Violin plots summarize six HD patients measured in two separate experimental runs. **(d)** Patient-specific outcome on motile function (left) and viability (right). Individual patients (n = six) are displayed separately. For clinical data see Table 2. P-value indicates significance level.

Next, we aimed to assess toxin removal by hemodialysis (HD) treatment. For this, we accessed serum from six ESKD patients (Table 2) that was drawn pre-HD and post-HD, respectively. A paired analysis via the HSTT allows an estimate whether HD procedure improves serum toxicity in the patient (Fig. 5a). Via the staining method, dead Eosin^+^ cells could be identified in pre-HD serum (Fig. 5b). In general, cells exposed to non-treated (pre-HD) serum had lower motility compared to cells exposed to treated (post-HD) serum (−8.6% ± 10.5%, p = 0.03) (Fig. 5c). Analogously, a significant decrease of viability of cells was detected when cells were exposed to pre-HD serum compared to post-HD serum (−17.8% ± 22.7%, p = 0.03) (Fig. 5c). In the motility analysis, sera from 5/6 patients were more toxic pre-HD compared to post-HD. Solely in HD-treated patient **#3** the opposite effect was observed. In the survival analysis, sera from 6/6 patients were more toxic pre-HD compared to post-HD (Fig. 5d). This indicates that either method discriminates pre versus post-HD serum. Further, we found that patients **#1** and **#5** were detected to have most toxic pre-HD serum compared to all other patients, as indicated by motility and survival readout, independently. Thus, we conclude that the HSTT can be applied to evaluate the presence of biologically active substances and further, the HSTT has the potential to assess the efficacy of toxin removal by treatments such as hemodialysis.

**Table 2:** Patient characteristics of the patients included in the study.

Patient	Gender	Age years	Disease	Children	HD- frequ. hrs per week	Dialyzer	HD/HDF	HDF liter	Vintage months	Urea pre-HD mmol/l	Urea post- HD mmol/l	Creatinine pre-HD µmol/l	Body weight kg	UF- volume liter
*P1*	man	71	DM type 1 (since 10 y age)	1	4 h × 4	FX1000	HDF	20	48	17.3	4.1	732	72	2.5
*P2*	man	72	Nephrosclerosis	0	4 h × 3	FX100	HDF	0	32	18.3	4.8	748	70	0.9
*P3*	man	85	Nephrosclerosis	2	4 h × 3	FX80	HD	0	52	21.0	5.8	634	86	1.3
*P4*	man	58	Hereditary polycystic kidney disease	3	4 h × 3	FX100	HD	0	55	18.2	4.8	874	54	2.5
*P5*	woman	77	Hydronephrosis, Kidney stones	4	3.5 h × 2	FX80	HD	0	31	12.5	3.2	564	74	0
*P6*	woman	50	DM type 1 (since 6 y age)	1	4 h × 2	FX1000	HDF	15	37	13.5	4.1	432	86	0

Details about hemodialysis procedure are shown. DM = diabetes mellitus, HD = hemodialysis, HDF = hemodiafiltration. UF = ultrafiltration. Urea in mmol/l. Creatinine in µmol/l. Vintage summarizes overall HD-time.

## Discussion

Via the human spermatozoa-based toxicity testing (HSTT) multiple substances of a toxic syndrome can be compared within one experimental set-up. Specifically, we propose motility testing of human spermatozoa as a highly sensitive tool to screen for *in-vivo* relevant uremic toxins. This is illustrated by a defined and dose-dependent motile response on uremic ultrafiltrate from ESKD patients, by a distinct single-substance response on previously denominated uremic toxins and eventually, by a sensitive detection whether serum from ESKD patients was detoxified by hemodialysis. In addition to this, survival analysis can be applied to identify very toxic substances and to define sublethal concentration of a toxin.

*In-vivo*, several organ systems are affected by the uremic state which requires experimental studies on various cell types *in-vitro*^2,39,41^. This has prompted strategies to systematically cover experimental studies on uremic toxin-induced effects^9^. However, experimental tools that compare multiple substances, in a serial manner, remain scarce. Via the HSTT, a broad number of substances can be studied and eventually, ranked according to toxicity. This may add guidance to identify single toxins within the complex nature of the uremic syndrome. Within the toxin screen, the effect of equimolar concentration of salt as control was not studied and should be mentioned as drawback of this study. Future studies evaluating the toxicity of single compounds in depth should include a salt-control condition.

The HSTT is based on a feasible cell counting technique that can be applied to standard lab procedures. We found *ex-vivo* accessed spermatozoa from healthy young men as highly functional starting material in our experiments. Recent improvements in freezing procedures of human spermatozoa for *in-vitro* fertilization^42^ will help to establish a standardized one donor-derived test kit for the HSTT, analogously. Based on our data, we propose healthy donors with viable cells that exert a baseline motility of 40 counts/minute as feasible donors for a kit.

Clinical symptoms that arise from a toxic syndrome - such as uremia - are vaguely understood^4^ and toxin removing strategies - such as hemodialysis – show inter-individual differences in improving uremic symptoms^43–45^. For now, KT/V_urea_-dosage is clinically applied to quantify dialysis time. We suggest that the HSTT may help to individually prescribe dialysis time. Hypothetically, this may result in improved symptom relief after treatment.

The data in the present study also imply that uremic substances may interfere with spermatozoon function *in-vivo*. Since male fertility is lowered in uremic men^26^, the HSTT may help to identify responsible substances for this effect. Eventually, this assay can be applied to compare semen from healthy versus semen from uremic men in further studies.

To this end, described effects by the HSST need to be confirmed by further cell models and HSST-identified toxins need to be confirmed by clinical studies. Here, all experiments were performed according to standardized guidelines published by the EUTox initiative^34^ that allows for comparative trials.

## Methods

### Donation and ethical approval

The Local Ethical Committee at Umeå University, Sweden approved this study (§268/01, dnr 01-255). In addition to this, we confirm that all experiments were performed in accordance with relevant regulations and guidelines for experimental studies on uremic toxins^41^. Semen samples were obtained after informed consent from 15 healthy human donors (21–30 years) without acute or chronic diseases, and medication free. Sexual continence before donation was not restricted. Serum samples were obtained from six hemodialysis patients after information and consent.

### Semen preparation

Human semen was accessed in a sterile container and directly incubated for 15 min at 37 °C for liquefaction^34^. Thereafter, semen was investigated for microscopic abnormalities (e.g. azoospermia) and to this end, no abnormal signs were observed (0/15). For the *in-vitro* set-up, semen samples were transferred to two ml polypropylene tubes (Sigma-Aldrich, Saint Louis, USA) and after transfer softly mixed for five sec to homogenize. All further incubation procedure was performed at 37° with minimum volume of 200 µl.

### Progressive motility investigation

#### Motility-based readout and controls

We transformed a previously described protocol^35^ to apply within an *in-vitro* culture readout with real-time analysis. Ten µl semen was injected into a disposable Bürker cell-counting chamber (DHC-B01, NanoEnTek, Digital Bio Technology Co., Inc., Seoul, Korea). Camera-integrated light microscopy (Carl Zeiss Microscopy, Jena, Germany) was performed with 400x magnification and 125–200 ms interval between two images. Recording time per sample was one minute and subsequently, images were used for post-experimental analysis. For this, a single line of 0.20 mm within the Bürker chamber was utilized as a crossing mark for counting (bold line in Fig. 1a). Every spermatozoon crossing this line was counted. If baseline motility exceeded 60 counts per minute, we diluted the semen with buffer (1 part semen to 1 part buffer). In most cases, 1:1 dilution was sufficient to proceed with the analysis. Thereby, we preserved seminal plasma in the system to maintain baseline stimulatory effect on the semen^35^. However, we did not apply (serum) albumin as control, as this may interfere with the metabolism of spermatozoa^46,47^. Instead, following buffers were tested for dilution: phosphate-buffered saline solution (PBS, Dulbecco’s Phosphate Buffered Saline, Thermo Fisher Scientific, USA), Krebs Ringer bicarbonate solution (KRB, Sigma-Aldrich, Saint Louis, USA), Ringer acetate solution (RA, Braun, Danderyd, Sweden), peritoneal dialysis solution with 1.36% glucose (PDS, Physioneal 35®, Baxter, Deerfield, USA) and saline (NaCl). After dilution, the mean starting motility was 40 cells/min (Suppl. Fig. 1a). PBS preserved motile capacity *in-vitro* (Suppl. Fig. 1b) and hence, PBS was applied for semen dilution and for substantial dilution of toxin reagents (Table 1). For analysis, Zeiss microscopy software (Zen 2012, Carl Zeiss Microscopy, Jena, Germany) was used.

#### Correction for time

Before incubation, baseline motility of two different samples was analyzed. This created a mean starting baseline. Consecutive measurements can be calculated as ratio of the baseline motility. We further adjusted for time if a sample was not correctly measured at the respective time-point. To adjust for delay, the decay of motility over time was calculated to receive a corrected motility count (Form. 1).

$${M}_{C}={M}_{B}+(C-B)\ast \frac{{M}_{B}-{M}_{A}}{A-B}$$

Formula 1. Time correction for motility analysis. To compare for one defined time point, motility counts can be adapted by this formula accounting for the linear decrease in function. (A: time point at the measurement; B: time point before the measurement; C: time point to be calculated for; all in minutes) (M_A_: motility at the measurement; M_B_: motility before the measurement; M_C_: motility to be calculated for; all in motile cells per minute).

### Viability analysis

A cell staining technique applied in human fertility testing was adapted to our method^28^. 30 µl semen was diluted (1:1) with cellular stains Eosin and Nigrosin (VitalStain™, Nidacon Int., Mölndal, Sweden) for 30 sec. A smear on a glass slide was performed and dried for at least 60 min. Oil immersion microscopy with 1000 × magnification was applied for analysis. Nigrosin served as enhancer of cellular contrast. Via light microscopy, Eosin^+^ dead could be identified by cytoplasmic incorporation of the red color (Fig. 4d). 100 cells were manually counted, and eventually, survival readout was calculated as the absolute number of Eosin^+^ dead per 100 cells. Calculation were performed six times within different fields of the slide.

### Preparation of uremic substances and patient serum

#### Uremic ultrafiltrate (UUF)

UUF was collected during hemodialysis with high flux membranes (FX80, Fresenius Medical Care, Bad Homburg, Germany) from five patients. Since ultrafiltration procedure (and no dialysis) was applied, UUF was not diluted. After filtration, UUF constitutes smaller sized substances in the same concentration present in blood^36^. Further, the ultrafiltrates were mixed and chromatographed to desalt, concentrate and fractionate the filtrate. For the chromatography, a preparative reversed phase C18 column was used as previously described^36^ which resulted into six fractions with increasing hydrophobicity (F1→F6). Eluted UUF was frozen at −80 degrees. In a next step, thawed UUF was applied 1:1 with semen (1:1 dilution of UUF; high concentration). In addition, thawed UUF was pre-diluted 1:8 with PBS and added 1:1 to the semen (1:16 dilution of UUF; low concentration). We replicated at least two non-toxic controls with cells from the same donation. For this, 100% PBS was applied.

#### Uremic toxin mix (UTM; Purine mixture)

A mixture of purine substances was prepared containing uric acid, xanthine, uridine and uracil. For dissolving, NaOH and further addition of Tris buffer was used to reach a final pH of 7.4 to 7.7^41^. The highest concentration of uremic toxins that was reported in uremic patients was applied^2,3,38,42^. For this, they are referred to as maximum concentration (C_max_). Before incubation, doubled C_max_-stock solutions were prepared to apply 1:1 dilution with the semen sample. This eventually resulted in a 1x C_max_ -concentration *in-vitro* for uric acid (147 µg/ml), xanthine (3.44 µg/ml), uridine (32.6 µg/ml) and uracil (0.45 µg/ml). To test for dose-dependency, UTM was applied in a low concentration (C_low_). For this, UTM was diluted 1:8 with PBS and applied 1:1 to the semen sample resulting in a 1:16 dilution of C_max_. For all toxins, we applied controls that were semen samples with a non-toxic medium (PBS). The control conditions were replicated four times and run in parallel to the toxic conditions.

#### Single uremic substances

47 uremic toxins (17 small water-soluble compounds (SWS), 16 protein bound (PB) and 14 middle molecules (MM)) were applied in C_max_^2,3,38,42^. For this, we pre-diluted the stock of toxins extensively with PBS to achieve 2 × C_max_-level. In a next step, we added the pre-diluted toxin 1:1 to the semen to achieve 1 × C_max_^2,3,38,42^ *in-vitro*. For individual C_max_ of substances see Table 1. The toxins are listed here in order of the number represented in Fig. 4a: (***SWS*** **1**: guanidine, **2**: malondialdehyde, **3**: 1-methyladenosine, **4**: taurocyamine, **5**: methylguanidine, **6**: cytidine, **7**: urea, **8**: orotidine, **9**: N-acetylarginine, **10**: uric acid, **11**: symmetric dimethyl arginine (SDMA), **12**: 1-methylguanosine, **13**: oxalate, **14**: uracil, **15**: hypoxanthine, **16**: guanidinoacetic acid, **17**: β-guanidinopropionic acid), (***PB*** **18**: leptin, **19**: kynurenine, **20**: kynurenic acid, **21**: methylglyoxal, **22**: phenyl sulfate, **23**: 2-methoxyresorcinol, **24**: 3-deoxyglucusone, **25**: homocysteine, **26**: p-OH-hippuric acid, **27**: putrescine, **28**: indoxyl glucuronide, **29**: p-cresyl glucuronide, **30**: phenyl glucuronide, **31**: indoxyl sulfate, **32**: p-cresol, **33**: p-cresyl sulfate), (***MM*** **34**: Interleukin (IL)-6, **35**: hyaluron, **36**: IL-1β, **37**: β-endorphin, **38**: neuropeptide-γ, **39**: Κ-Ig light chain, **40**: retinol binding protein, **41**: adrenomedullin, **42**: tumor necrosis factor(TNF)-α, **43**: endothelin, **44**: atrial natriuretic peptide (ANP), **45**: Lambda-Ig light chain, **46**: complement factor D, **47**: cholecystokinine). P-cresyl sulfate and phenyl sulfate were synthesized according to Feigenbaum and Neuberg as potassium salt^48^. P-cresyl glucuronide was synthesized from glucuronyl-trichloracetimidate and p-cresol using a protocol adapted from Van der Eycken and colleagues^49^. All remaining substances including indoxyl-β-D-glucuronide cyclohexylammonium salt, indoxyl sulfate potassium salt, potassium chloride, ammonium chloride and cyclohexammonium were purchased from Sigma-Aldrich Co, St. Louis, MO, USA.

#### Drug

Soluble furosemide (Nycomed, So4lna, Sweden) was applied in concentration calculated to the following formula: *(medication dose given per day: 1 mg)*/[*0.6 (distribution volume)* × *body weight: 100 kg]*. This resulted in an estimated *in-vitro* concentration of 166.7 µg/ml that was applied for *in-vitro* culture. Similar plasma levels were observed when furosemide was applied to ESKD patients^50^. To test for *in-vitro* dose-dependency, furosemide was further applied in a 1:4 dilution (1 × drug to 4 × PBS; C_low_) and additionally, applied in a 10x concentration of C_norm_ as C_high_. This resulted into *in-vitro* concentrations: C_high_: 1666,7 µg/ml, C_norm_: 166,7 µg/ml, C_low_: 41,7 µg/ml.

### Patient serum

Whole blood samples of patients were centrifuged at 1000 g for five minutes at room temperature in serum separating tubes and subsequently, serum was transferred into a new vial. Incubation of serum was performed 1:1 with *ex-vivo* gained semen.

### Statistics

Non-parametric paired comparisons were used by Wilcoxon analysis. Mann Whitney test was used for group comparison. Tests were calculated via IBM statistic software SPSS version 22 and a p-value of <0.05 was considered to be significant. Mean and standard deviations are presented throughout, unless otherwise indicated. R 3.5.1 coding software was applied to create the heat map. Overleaf v2 software was used to create initial graphical display of the mathematical formula. For all remaining graphs, Graph pad prism version 8.0.2 was used.

## Supplementary information

Supplementary Figures 1 and 2

## Data Availability

The authors declare that all data supporting the findings of this study are available within the paper.
