# Supplementary material for: An in-vitro assay using human spermatozoa to detect toxicity of biologically active substances
Source: Sci Rep. 2019 Oct 10;9:14525. doi: 10.1038/s41598-019-50929-z (PMC6787250; doi:10.1038/s41598-019-50929-z)
Supplement: Supplementary file 1 — Supplementary Figures 1 and 2 [file 41598_2019_50929_MOESM1_ESM.pdf]

Supplementary Information

To An *in-vitro* assay using human spermatozoa to detect toxicity of biologically active substances

Tino Vollmer, Börje Ljungberg, Vera Jankowski, Joachim Jankowski, Griet Glorieux, Bernd G Stegmayr

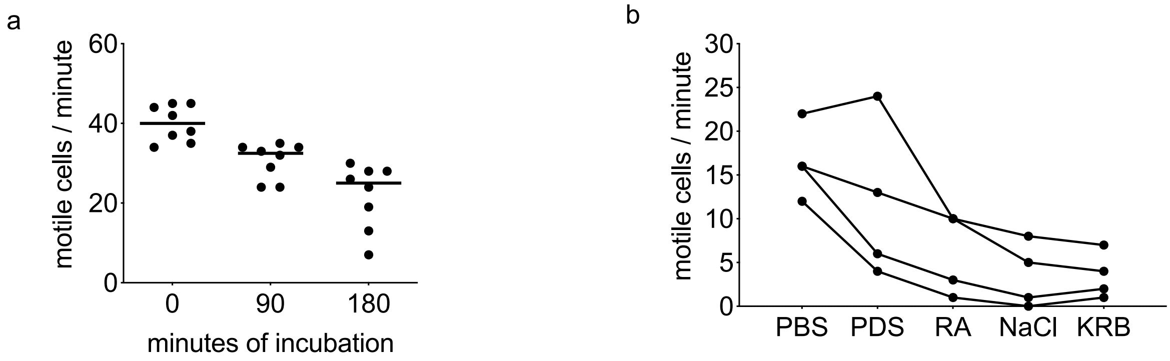

Supplementary Figure 1. Motility analysis establishment. **(a)** Setting up motility counts per time. Four donors with two independent experiments each displayed. Dot represents one sample. **(b)** Buffer establishment for motility analysis. Phosphate buffered saline (PBS), Krebs Ringer bicarbonat solution (KRB), Ringer acetate solution (RA), peritoneal dialysis solution with 1.36% glucose (PDS) and saline (NaCl) compared. Incubation performed for 120 minutes. Two donors with two independent experiments each shown.

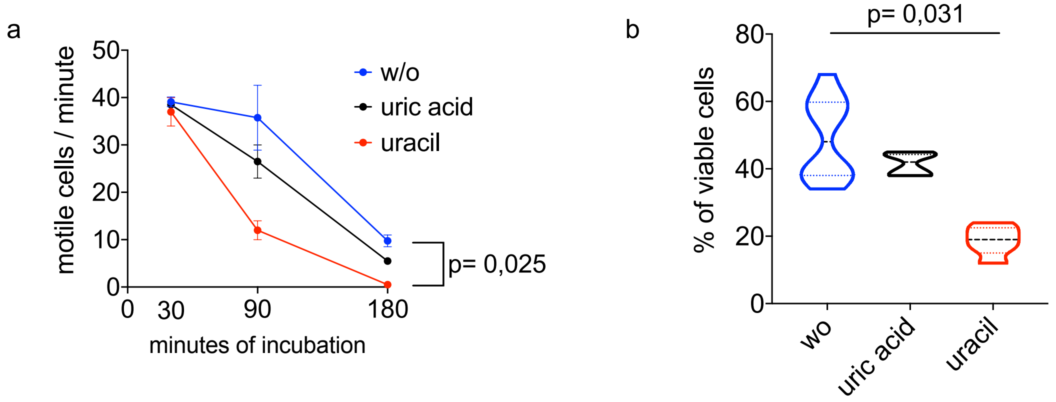

Supplementary Figure 2. Toxicity testing of compounds within the uremic toxin mix. Two substances tested for toxicity within the uremic toxin mix. Uracil and Uric acid. **(a)** Motility analysis of two experimental rounds on cells from two donors. **(c)** Violin plots show survival analysis of six experimental rounds on cells from two donors. Incubation time was 180 minutes. P-value indicates significance level.
